# Liquid metal integrated PU/CNT fibrous membrane for human health monitoring

**DOI:** 10.3389/fbioe.2023.1169411

**Published:** 2023-03-31

**Authors:** Mei-Xi Li, Da-Yong Wu, Rong-Yu Tang, Si-Yuan Zhou, Wei-Hua Liang, Jing Liu, Lei Li

**Affiliations:** ^1^ Technical Institute of Physics and Chemistry, Chinese Academy of Sciences, Beijing, China; ^2^ Key Laboratory of Cryogenics, Technical Institute of Physics and Chemistry, Chinese Academy of Sciences, Beijing, China; ^3^ The State Key Laboratory on Integrated Optoelectronics, Institute of Semiconductors, Chinese Academy of Sciences, Beijing, China; ^4^ School of Chemical Sciences, University of Chinese Academy of Sciences, Beijing, China

**Keywords:** electrospinning, liquid metal, carbon nanotubes, multiscale conductive structure, human health monitoring

## Abstract

Wearable flexible sensors are widely used in several applications such as physiological monitoring, electronic skin, and telemedicine. Typically, flexible sensors that are made of elastomeric thin-films lack sufficient permeability, which leads to skin inflammation, and more importantly, affects signal detection and consequently, reduces the sensitivity of the sensor. In this study, we designed a flexible nanofibrous membrane with a high air permeability (6.10 mm/s), which could be effectively used to monitor human motion signals and physiological signals. More specifically, a flexible membrane with a point (liquid metal nanoparticles)-line (carbon nanotubes)-plane (liquid metal thin-film) multiscale conductive structure was fabricated by combining liquid metal (LM) and carbon nanotubes (CNTs) with a polyurethane (PU) nanofibrous membrane. Interestingly, the excellent conductivity and fluidity of the liquid metal enhanced the sensitivity and stability of the membrane. More precisely, the gauge factor (GF) values of the membrane is 3.0 at 50% strain and 14.0 at 400% strain, which corresponds to a high strain sensitivity within the whole range of deformation. Additionally, the proposed membrane has good mechanical properties with an elongation at a break of 490% and a tensile strength of 12 MPa. Furthermore, the flexible membrane exhibits good biocompatibility and can efficiently monitor human health signals, thereby indicating potential for application in the field of wearable electronic devices.

## 1 Introduction

In recent years, with the development of mobile healthcare, intelligent wearable sensor devices have been gradually adopted in human health monitoring applications (Gong et al., 2022), such as voice sensors for the artificial throats (Lee et al., 2021); flexible humidity sensors for real-time monitoring of sleep conditions (Honda et al., 2022); and a visual electrocardiogram (ECG) synchronization monitor (Luo et al., 2021). Specifically, users can effectively complete real-time healthcare and on-demand treatment at home by wearing these sensor devices, which greatly improves treatment efficiency and reduces medical costs. Generally, wearable sensor devices are usually required to be attached to clothing or the skin. More so, to adapt to the human body, the materials utilized for wearable devices should be flexible, deformable, malleable, and conformable (Zou et al., 2019). More importantly, these materials need to ensure the sensitivity of detection (Bubnova, 2017; Lin et al., 2020).

Typically, conductive polymer composites (CPCs) are made by dispersing conductive fillers in the polymer matrix through blending or dip-coating processes (Wang et al., 2017; Pan et al., 2020). After the deformation of the CPCs, the microstructure change of the composite leads to a change in electrical resistance, which produces an altered electrical signal (Amjadi et al., 2016). Particularly, this characteristic makes CPCs widely used in the field of sensing. Further, various carbon materials are often used as conductive fillers, such as carbon nanotubes (CNTs), carbon black (CB), graphene, carbon nanofibers (CNF) (Wang et al., 2021). Besides, owing to the large aspect ratio and excellent electrical conductivity, CNTs can effectively improve the mechanical properties and sensitivity of CPCs (Zhou et al., 2020; Kanoun et al., 2021). In addition, CNTs have good biocompatibility, which makes them suitable for the field of wearable devices (Yang et al., 2021). For example, in a stretchable strain sensor, CNTs were diffused into polydimethylsiloxane (PDMS) by a simple swelling/permeating method to obtain composite conductive materials with excellent flexibility and high sensitivity (Zhang et al., 2019). In another approach, the CNTs were doped into graphene-PDMS to fill the gaps between the adjacent graphene, which significantly improved the electrical conductivity and mechanical properties of graphene-PDMS composites (Ko et al., 2021). However, the solid CNTs conductive fillers usually separate and break during stretching, bending and large deformation, This results in the reduction of conductivity and affects the continuity and accuracy of the signal (Yu et al., 2020).

Gallium-based liquid metals (LMs) are metal alloys with a low melting point and high conductivity (*σ* = 3.4 × 10^4^ S/cm); good fluidity (viscosity ≈2 MPaS); and high deformability (Zheng et al., 2019; Hang et al., 2021; Zhang et al., 2022). At present, LM has shown great utility value in soft robots (Wang et al., 2018; Zheng et al., 2022), biomedicine (Feig et al., 2022; Gao et al., 2022), energy management (Zuraiqi et al., 2020; Zhang et al., 2021), flexible electronics (Liang et al., 2022), *etc.* Besides, LM is suitable for manufacturing stretchable conductors because it can easily deform with the stretching and bending of flexible substrates (Wang et al., 2023). Additionally, LM has been successfully introduced into hydrophilic polymer networks as soft fillers for sensors (Peng et al., 2019). The use of LM as a “binder” of solid conductive fillers is expected to improve the conductivity reduction of CPCs due to tensile deformation. For example, Andrew’s team used LM as the connection point of the printed CNTs channels, making the on-current of the device reach 150 μA/mm (Andrews et al., 2018). In addition, He et al. used LM/CNTs as mixed fillers and encapsulated them into PDMS to prepare a strain sensor that may be deployed to detect the movement of finger joints (Pan et al., 2020). Specifically, the sensor has remarkably robust stability of resistance stability under 15%–60% strain. Further, Wu et al. introduced CNTs bridged LM composite into the hydrogel to obtain a strain sensor with excellent conductivity and transparency, which could be implanted into the heart of Chinese sturgeon to monitor its health status (Sun et al., 2023).

Although the above-mentioned studies have been able to obtain high-performance stretchable sensors, they often ignore the permeability. Particularly, the impermeable sensors may cause skin inflammation and affect overall signal detection (Shen et al., 2021). The flexible nanofibrous membrane prepared by electrospinning technology has a large number of pores and high permeability, which can provide sufficient space for gas exchange. Moreover, the membrane with a high specific surface area may contain a large number of conductive fillers, which become an ideal substrate for flexible wearable sensors (Liman et al., 2021). Li et al. printed the LM and silver flake on the nanofiber-based triboelectric nanogenerator (TENG) with high triboelectric output, which is expected to be used in wearable power sources and electronic skin (Li et al., 2020). Wang et al. coated LM droplets onto an electrospun TPU fiber film and following this, suspended the LM between the TPU fibers through a mechanical stretching process to obtain a flexible sensor with a working range between 0% and 200% and a gauge factor (GF) of 0.2 (Wang et al., 2022). Hao et al. adopted the stencil printing method to pattern liquid metal on the hydrogel substrate to prepare hydrogel-based soft electronics with a GF of 1.42 at 630% strain (Hao et al., 2021). In particular, printing LM directly on the surface of the fiber membrane could provide good electrical conductivity for composite materials, but the detection sensitivity was insufficient. Specifically, the quantum size effects of nanomaterials can significantly improve the sensitivity of the sensor (Wang et al., 2016). Therefore, the introduction of multiscale conductive materials into the system can be attempted to improve the detection sensitivity of the sensor.

In this work, liquid metal nanoparticles (LMNPs) were dispersed into polyurethane (PU) solution, and then the solution was subsequently made into a flexible fibrous membrane by electrospinning. After this, the CNTs were adhered on the nanofiber by ultrasonic treatment to form a conductive membrane. Finally, a layer of LM was scraped on the surface of the membrane to obtain a flexible nanofibrous membrane with a point (LMNPs)-line (CNTs)-plane (LM thin-film) multiscale conductive structure. The cycling stability and detection sensitivity of the membrane during deformation were studied.

## 2 Materials and methods

### 2.1 Materials

Polyurethane (PU, SKU 430218) was purchased from Sigma-Aldrich (St. Louis, MO, United States). N,N-dimethylformamide (DMF, AR) and tetrahydrofuran (THF, AR) were purchased from Beijing Chemical Works (Beijing, China). Gallium (99.99%) and indium (99.995%) were purchased from Shanxi Zhaofeng Gallium Co. Ltd. (China). Multi-walled carbon nanotube (CNTs, Purity >95%) and dispersant for CNTs aqueous solutions (XFZ20, surfactant content 90%) were purchased from Chengdu Zhongke Times Nano Energy Tech Co., Ltd. Dulbecco’s modified Eagle medium (DMEM, REF 11995-065), fetal bovine serum (FBS, REF 10099-141), penicillin/streptomycin (P/S, REF 15140-122) solution, and phosphate buffered saline (PBS, REF C10010500BT) were purchased from Gibco (Grand Island, NY, United States of America). Cell Counting Kit-8 (CCK-8) and Calcein-AM/PI (CA1630) were purchased from Beijing Solarbio Science Technology Co., Ltd. Unless otherwise specified, all raw materials were not further purified before use.

### 2.2 Preparation

#### 2.2.1 Preparation of the GaIn alloy

The gallium and indium were weighted with a mass ratio of 75.5: 24.5 and added in a beaker, then heated and stirred at 80°C for 30 min to make the eutectic gallium–indium alloy (EGaIn, melting point: 15.5°C).

#### 2.2.2 Electrospinning solution

EGaIn with 1% volume was added into DMF/THF mixed solvent (1/1, v/v) and sonicated for 30 min. Then PU (9 g) was dissolved in the mixed solvent (100 mL) to obtain a 9% w/v LM@PU solution.

#### 2.2.3 Preparation of the LM@PU/CNT/LM membrane

**FIGURE 1:**
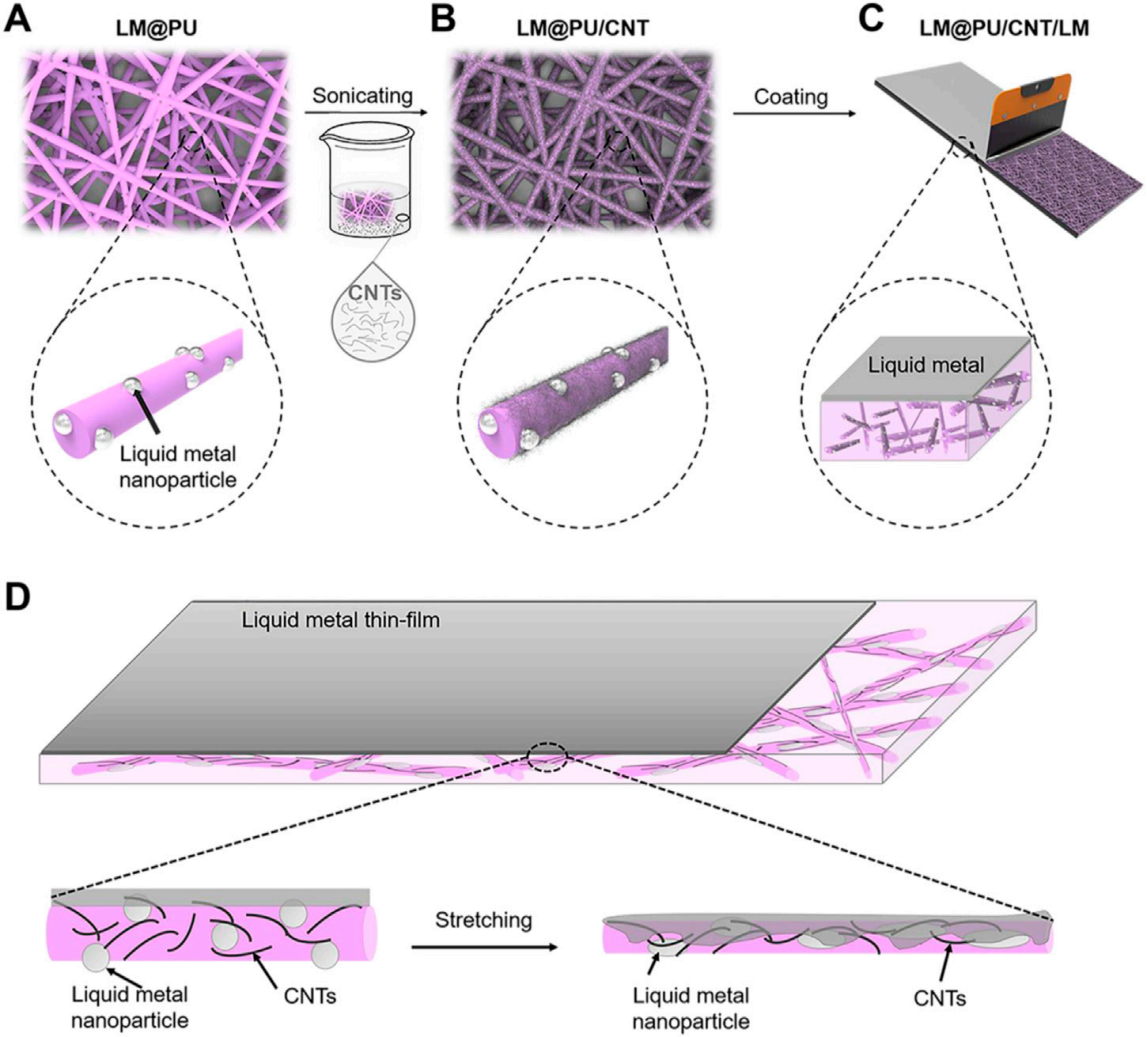
Schematic diagram of the **(A)** LM@PU, **(B)** LM@PU/CNT, **(C)** LM@PU/CNT/LM membranes preparation, and **(D)** the strain-sensing mechanism of the synergistic effect of multiscale conductive structure.

The LM@PU/CNT/LM membrane preparation is illustrated schematically in Figure 1. First, the LM@PU solution was injected into the electrospinning device (Li M. X. et al., 2021) to prepare the nanofiber membrane (labeled as LM@PU membrane). The electrospinning parameters were set as follows: the temperature was 25°C and the related humidity was 30%. The LM@PU solution was drove by a syringe pump with the flow rate of 3.0 mL h^−1^ for 5 h. The receiving distance and the output of the direct current power supply were set as 15 cm and +12 kV, respectively. The thickness of LM@PU membrane is 116.6 ± 1.8 μm. Under the same experimental conditions, 9w/v% PU solution was electrospun to obtain a PU membrane with a thickness of 113.6 ± 1.9 μm. Subsequently, CNTs (1 g) and dispersant (200 mg) were added into 200 mL deionized water, followed by ultrasonic treatment 60 min to obtain CNTs dispersion. The LM@PU membrane was immersed in the CNTs dispersion and sonicated for 5 min, and then put it into a vacuum oven for drying at room temperature to obtain the membrane coated with CNTs (labeled as LM@PU/CNT membrane). Finally, the liquid metal (mass loading 2.3 mg/cm^2^) was dropped onto the LM@PU/CNT membrane, and then a scraper was used to coat the liquid metal evenly on the LM@PU/CNT membrane (labeled as LM@PU/CNT/LM membrane). The PU membrane was treated in the same way and was labeled as PU/LM membrane. For the application of flexible sensor, a layer of LM@PU mat was electrospun onto the surface of the LM@PU/CNT/LM membrane to serve as the encapsulation layer.

### 2.3 Characterization

The surface morphology of the samples was observed *via* field emission scanning electron microscopy (FE-SEM, S-4800, Hitachi, Japan) with an accelerating voltage of 10 kV. Energy dispersive spectroscopy (EDS) was used to analyse the elemental distribution. The mechanical properties of the samples were tested by a universal testing machine (INSTRON 5966, Canton, MA) at a strain rate of 1 mm/min. The air permeability and moisture permeability of the samples were tested by the automatic breathability tester (YG461E, Zhejiang Sangongjiang Instrument Co., Ltd.) and the fabric moisture permeability tester (YG601H-II, Ningbo Textile Instrument Factory), respectively.

### 2.4 Antibacterial and biocompatibility tests

LM@PU/CNT/LM and PU membranes were cut into 13 mm-diameter circles and sterilized by ultraviolet irradiation for 30 min.

To evaluate the antibacterial properties of the samples, *E. Coli* strains (ATCC 25922) were used to perform the experiments. E. *coli* cells were grown on Luria-Bertani (LB) agar plates at 37°C for 12–16 h. Additionally, monoclonal colonies were inoculated in an LB medium with shaking at 37°C. Bacterial culture was harvested at the exponential phase and the number of live bacteria was determined by the spread-plate method. Bacterial culture was diluted to approximately 2 × 10^5^ colony-forming unit (CFU) mL^−1^ in PBS for the antibacterial experiment. The sterilized membranes were placed in a 24-well plate, and 100 μL of the bacterial solution was added onto the membranes. The 24-well plate was then placed in an incubator at 37°C. After 6 and 12 h, the viable cells were determined by the spread-plate method. The bacteria cultured without membrane were used as the control group.

L929 Mouse Fibroblast cells (ATCC, US) were adopted to evaluate the biocompatibility of samples. L929 cells were cultured in complete medium supplemented with 1% P/S and 10% FBS. Precisely, the LM@PU/CNT/LM and PU membranes were immersed in culture medium for 24 h, respectively. 6 × 10^4^ L929 cells in 1 mL complete medium were seeded in a 24-well plate and incubated in the cell incubator of 37 °C and 5% CO_2_. After 24 h of incubation, the complete medium in the 24-well plate was removed, and 1 mL membrane-soaked medium was added in the 24-well plate. After 72 h of incubation, Live/Dead fluorescence staining and CCK-8 assays were used to quantify the cell viability. L929 cells cultured in complete medium were used as the control group.

The skin compatible performance of the LM@PU/CNT/LM membrane was evaluated on the Japanese white rabbit skin according to ISO 10993-10:2010 (E) standard. Specifically, all procedures of *in vivo* animal experiments were approved by the Technical Institute of Physics and Chemistry, Chinese Academy of Sciences (No. LHDW-22044-1). Four samples (negative control: medical gauze; commercial electrodes; LM@PU/CNT/LM membrane; Positive control: medical gauze rinsed with saturated sodium dodecyl sulfonate solution) were cut into 2.5 × 2.5 cm squares and attached to the skin of the rabbit. The samples were removed after 72 h. The skin was photographed at 0, 24, 48, and 72 h.

### 2.5 Measurements

The electromechanical properties of the LM@PU/CNT/LM and PU/LM membranes were tested by a digital nano voltmeter (Keysight 34420A). The PU/LM、LM@PU/LM, and LM@PU/CNT/LM membranes were respectively connected in series with LED in the circuit to test the conductivity of the membranes in the direction of thickness. In the circuit, the back side of the membrane (without liquid metal thin-film) was connected to the LED and the front side of the membrane (with liquid metal thin-film) was connected to the wire. The LM@PU/CNT/LM membrane and commercial electrodes (HRKT-RN55, Beijing Huarun Kangtai High-tech Development Institute) were attached to near the wrist and recorded ECG signals by OmniPlex (Plexon Inc.). Additionally, the LM@PU/CNT/LM membrane and commercial electrodes were attached to the flexor carpi ulnaris and recorded the electromyogram (EMG) signals during the muscle extension and contraction states by OmniPlex. The signals were analyzed using MATLAB software. The LM@PU/CNT/LM flexible sensor was adhered to the human body to detect the motion signals. The electrochemical workstation (CHI660E, Shanghai Chenhua Instrument Co., Ltd.) was used to record the electrical signals. The volunteers involved in the experiments have signed informed consent.

## 3 Results and discussion

### 3.1 Structure and morphology

**FIGURE 2:**
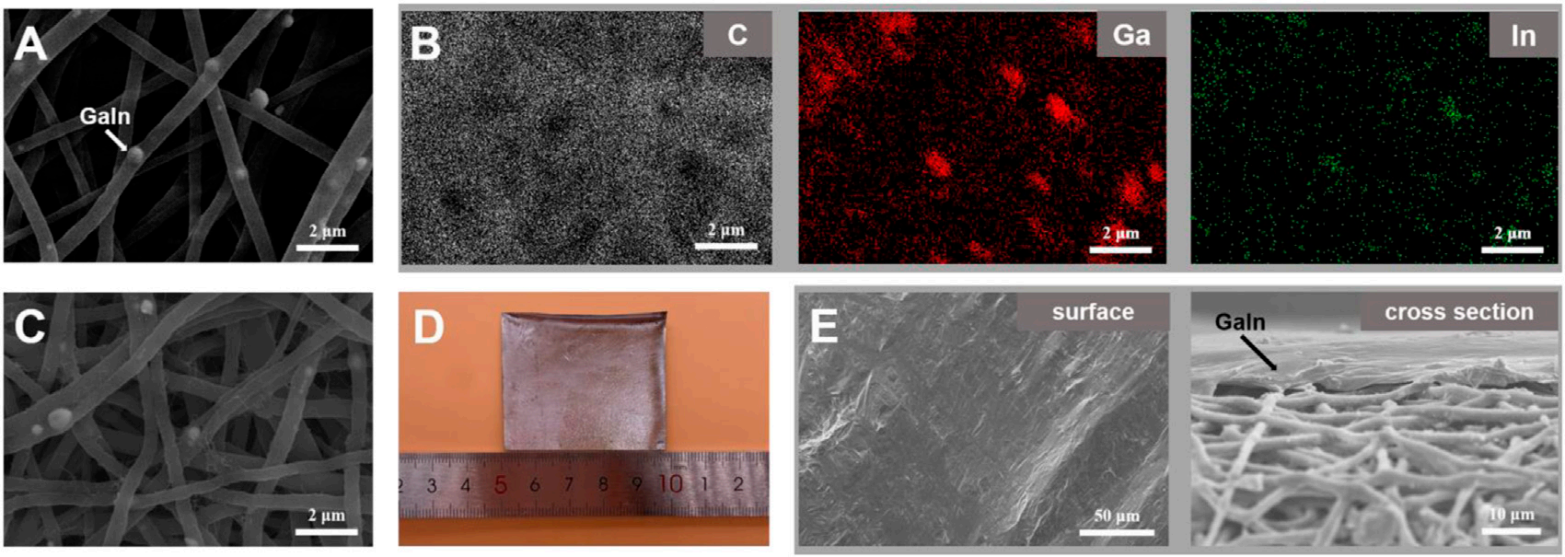
**(A)** SEM image of the LM@PU membrane. **(B)** The corresponding elemental mapping of the LM@PU membrane. **(C)** SEM image of the LM@PU/CNT membrane. **(D)** Digital image of the LM@PU/CNT/LM membrane. **(E)** SEM images of the surface and cross section of the LM@PU/CNT/LM membrane.

Figures 2A,B are the SEM image and corresponding elemental mapping of the LM@PU membrane. The C element is from the polyurethane. The bright spots (488.1 ± 169 nm in diameter) embedded in the fibers are confirmed to be Ga and In. Figure 2C is the SEM image of the LM@PU/CNT membrane. After ultrasonic treatment with CNTs dispersion, the LM@PU nanofiber was wrapped with CNTs. Figures 2D,E are the digital and SEM images of the LM@PU/CNT/LM membrane. It can be observed that the surface of the membrane was coated with the dense LM thin-film and presented a metallic lustre, indicating that LM was evenly scraped on the LM@PU/CNT membrane.

### 3.2 Mechanical properties

**FIGURE 3:**
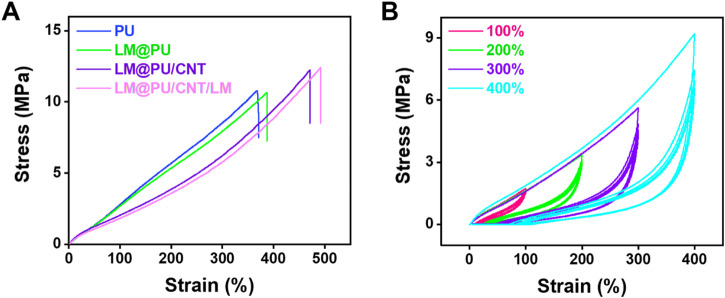
**(A)** The stress-strain curves of PU, LM@PU, LM@PU/CNT, and LM@PU/CNT/LM membrane. **(B)** The stress-strain curves of LM@PU/CNT/LM membrane under five cycles of loading-unloading.

Figure 3A shows the mechanical properties of the PU, LM@PU, LM@PU/CNT, and LM@PU/CNT/LM membrane. It can be found that the PU membrane possesses an elongation at a break of 390% and a tensile strength of 10 MPa, while the incorporation of LM and CNTs improves the elongation at breaks and tensile strength of the LM@PU/CNT/LM membrane to 490% (27% increase) and 12 MPa (20% increase). This is related to the addition of the two conductive fillers—LMNPs and CNTs. Furthermore, the mechanical properties of the membrane are greatly enhanced by adding CNTs to the membrane.

The materials of flexible sensor should have good mechanical properties to adapt to a variety of applications. Electrospinning is an efficient method for preparing polymer nanofibers (Awad et al., 2018; Xue et al., 2019). The nanofibers obtained by electrospinning are mostly distributed randomly, and their orientation and crystallinity are generally inadequate (Rim et al., 2013). Thus, it is often necessary to improve the mechanical properties of nanofibers. More so, compared with rigid filler particles, (Chen et al., 2019; Pang et al., 2019), the embedded liquid metal nanoparticles are highly deformable and can be stretched as the surrounding elastomer deforming without inducing internal stress concentrations and introducing significant mechanical resistance (Malakooti et al., 2019). Additionally, CNTs are commonly used as reinforcement materials for polymers due to the high aspect ratio (lengths of up to mm and diameters ranging from 1 to 100 nm) and excellent mechanical properties (Huang et al., 2021). Interestingly, when CNTs are combined with fibers, the stress of the fibrous matrix can be transferred to the CNTs, thereby improving the mechanical properties of the electrospinning membrane (Wang et al., 2019).

The reproducibility of the LM@PU/CNT/LM membrane was investigated through cyclic loading-unloading experiments. Figure 3B shows the stress-strain curves of the LM@PU/CNT/LM membrane within five cycles under different strains (100%, 200%, 300%, and 400%). It can be seen that the LM@PU/CNT/LM membrane has good resilience during the deformation, which satisfies the stable response of flexible strain sensors. During the initial loading cycle process, the rearrangement and stretching of the disordered fibers may be accompanied by fiber breakage or energy dissipation by the friction of highly entangled molecular chains (Zhou et al., 2022). Therefore, the elastic modulus of the first loading cycle is higher than that of the following loading cycles. The stress-strain curves in the subsequent loading cycles are highly coincident, implying the self-recovery ability of the elastomer upon release (Liang et al., 2021).

### 3.3 Strain-sensing performance

**FIGURE 4:**
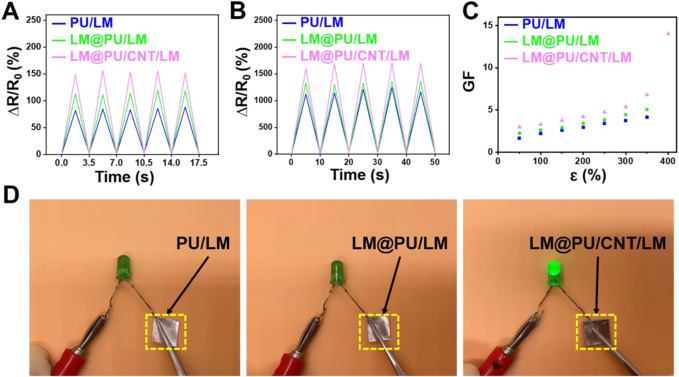
Relative resistance changes of membranes at **(A)** 50% deformation and **(B)** 300% deformation under five cycles of loading-unloading. **(C)** Plot of the GF as a function of the strain of membranes. **(D)** Comparison of the conductivity of the PU/LM, LM@PU/LM, and LM@PU/CNT/LM membrane in the direction of thickness.

The LM@PU/CNT/LM membrane was stretched to different lengths and the resistance was measured to evaluate the strain-sensing performance. As illustrated in Figures 4A,B, the PU/LM, LM@PU/LM, and LM@PU/CNT/LM membranes exhibited repeatable and stable electrical response at 50% strain (small deformation) and 300% strain (large deformation) under five cycles of loading-unloading. The relative resistance changes (∆R/R_0_) of the PU/LM, LM@PU/LM, and LM@PU/CNT/LM membranes at 50% strain were 82%, 113%, and 149%, respectively. LMNPs could significantly improve the sensing performance of the flexible membrane during small deformation. Particularly, when the strain was 300%, the ∆R/R_0_ of the PU/LM, LM@PU/LM, and LM@PU/CNT/LM membranes were 1,123%, 1,332%, and 1,605%, respectively. GF is a common parameter representing the sensitivity of the sensor to strain. It is defined as the ratio of the relative change of the output signal to the applied strain and can be calculated by the following formula:

$$GF=\frac{\left(\frac{∆R}{R_{0}}\right)}{\varepsilon }$$ (1)

Where Δ*R* is the change in the resistance, *R*_*0*_ is the original resistance, and *ε* is the applied strain. The GF values of the LM@PU/CNT/LM membrane are higher than that of the PU/LM and LM@PU/LM membranes under the same deformation (Figure 4C). The GF values of the LM@PU/CNT/LM membrane is 3.0 at 50% strain and 14.0 at 400% strain, which is much higher than the sensor obtained by directly coating the liquid metal onto the fibrous membrane (Wang et al., 2022). The high sensitivity of the LM@PU/CNT/LM membrane may be attributed to the point (LMNPs)-line (CNTs)-plane (LM thin-film) multiscale conductive structure of the LM@PU/CNT/LM membrane. The liquid metal exposed to air will form an oxide layer on the surface immediately. During the stretching process, the oxide layer of the liquid metal thin-film on the surface of the flexible membrane will fracture with deformation. The liquid metal nanoparticles dispersed in the fibers under the strain can also break the outer oxide shell to expose GaIn-alloy in the inner layer. When stress is applied to the membrane, the fibers extend along the stretching direction and the diameter of the fibers decreases. As a result, the LMNPs and the CNTs on the fibers are deformed or even broken, and the compact LM thin-film broke up into a porous structure (Ma et al., 2021). Along with the change of position between the fibers, the conductive network was damaged and new conductive pathways were formed (Wang et al., 2019). It is reasonable that a larger deformation causes greater resistance changes. When the applied stress was gradually released, the fibers tended to return to their original state, while the LMNPs, CNTs, and LM thin-film also tended to return to their initial state thus causing the conductive network to repair. The synergistic effect of multiscale conductive structure makes the LM@PU/CNT/LM membrane exhibit higher sensing performance than the other two membranes.

**FIGURE 5:**
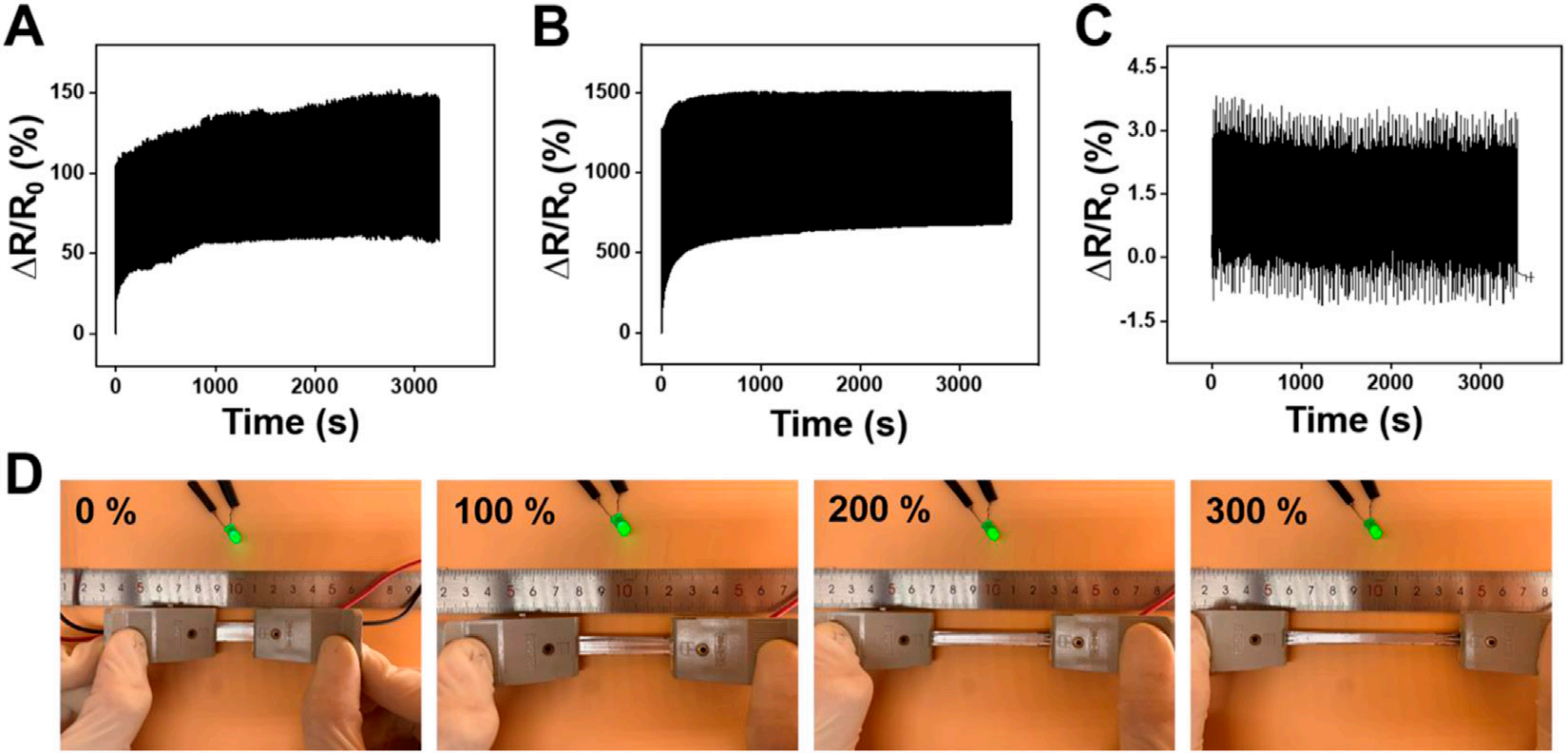
Relative resistance changes of the LM@PU/CNT/LM membrane under cyclic tensile test at **(A)** 50% strain and **(B)** 300% strain. **(C)** Relative resistance changes of the LM@PU/CNT/LM membrane in bending fatigue test. **(D)** LED brightness as a function of strain connected with the LM@PU/CNT/LM membrane.

Further, the durability and stability performance of the LM@PU/CNT/LM membrane under different deformations are shown in Figures 5A–C. The LM@PU/CNT/LM membrane exhibited good repeatability and stability performance during the tensile test and bending fatigue test, which guaranteed the application in different fields. As shown in Figures 5A,B, the relative resistance changes of the LM@PU/CNT/LM membrane had a minor irreversible change during the initial cycles, and then stabilized in the subsequent cycles. During the initial cycles, the conductive pathways between the liquid metal and CNTs were unstable. As the stretch-relax cycle proceeds, the connection between liquid metal and CNTs was closer, and the conductive pathways tend to be stable as well. In addition, there was a certain hysteresis effect in the initial stage of stretch-relax, which might also affect the resistance response of the LM@PU/CNT/LM membrane. Flexible sensors must have stable electrical conductivity during the deformation. To have an insight into the stable electrical conductivity of the LM@PU/CNT/LM membrane, a light-emitting diode (LED) was connected in series with the membrane in a circuit. As shown in Figure 5D, with the extension of the membrane from 0% to 300%, the brightness of the LED is almost constant, indicating that the membrane has stable electrical conductivity, even in the large deformation. In addition, the conductivity between the two sides of the membrane was tested by connecting the two ends of the LED on the front-side and back-side of the membranes, respectively (Figure 4D). The results show that the LED is “on” when connected to the LM@PU/CNT/LM membrane, but is “off” when connected to the PU/LM and LM@PU/LM membranes, indicating that the thickness direction of the LM@PU/CNT/LM membrane was conductive. The conductivity (σ) of the membranes can be approximately calculated by the following formula:

$$\sigma =\frac{L}{W\times T\times R}$$ (2)

where *L*, *W*, and *T* are the length, width, and thickness of the conducting layer, respectively, *R* is the measured electrical resistance. The conductivity of the PU/LM, LM@PU/LM, and LM@PU/CNT/LM membranes are 6 × 10^3^ S cm^-1^, 8 × 10^3^ S cm^-1^, and 1 × 10^4^ S cm^-1^, respectively. Therefore, the addition of LMNPs and CNTs significantly improved the conductivity of the LM@PU/CNT/LM composite membrane. The CNTs coated on the fibers were tightly connected to form conductive pathways between the fibers and as a result, enhanced the conductivity of the whole membrane. During the process of continuous stretching, the liquid metal thin-film on the surface of the membrane gradually infiltrates into the membrane and hung on the conductive fibers. Due to their excellent electrical conductivity and high aspect ratio, CNTs can easily form conductive pathways in polymers and connect the LMNPs embedded in the fibers and LM thin-film on the surface of the membrane. Thus, a closer connection of the LMNPs-CNTs-LM conductive pathway is formed, which greatly maintains the stability of the conductive network during the stretching process.

### 3.4 Biocompatibility and antibacterial properties

**FIGURE 6:**
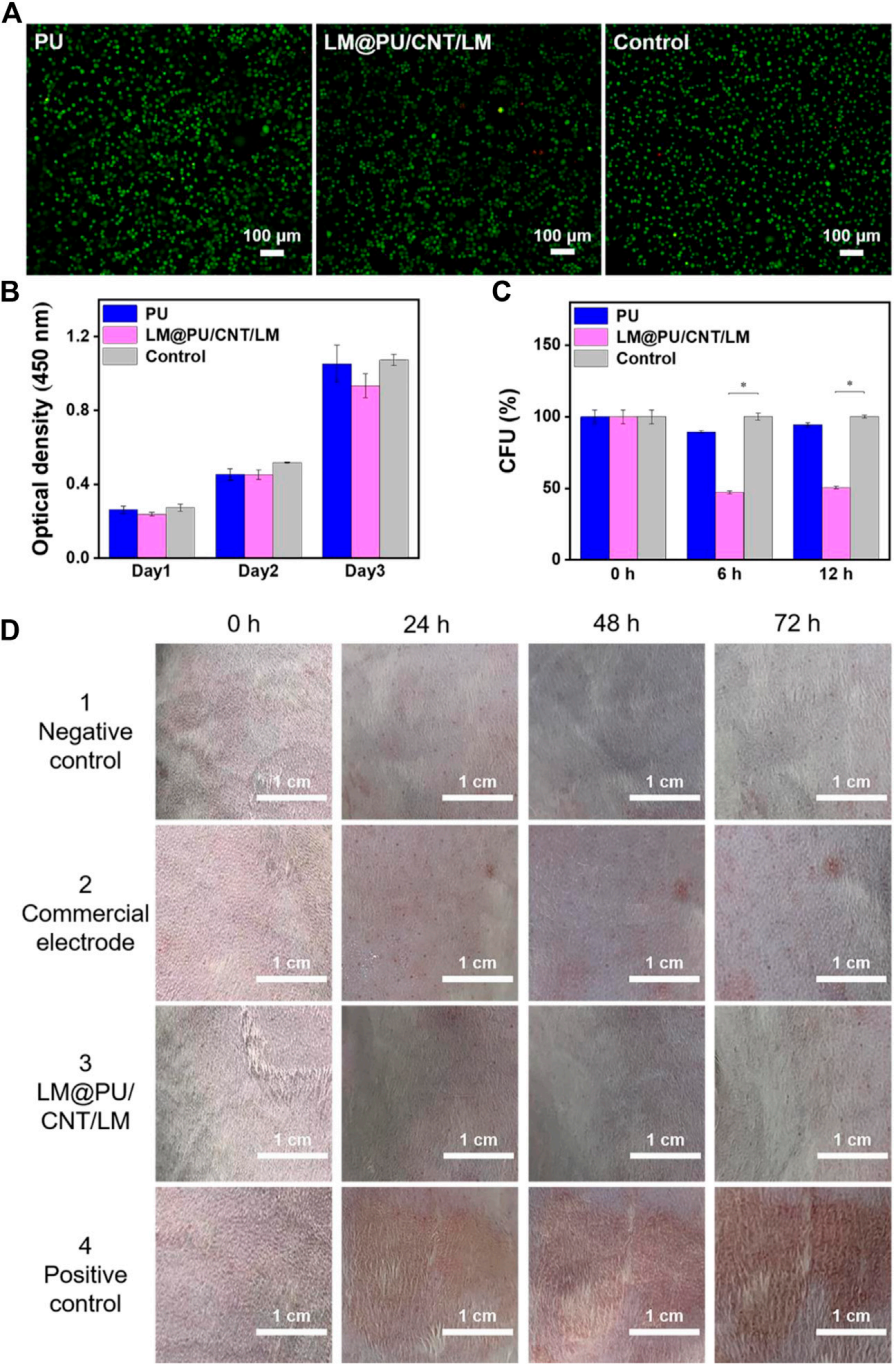
**(A)** Live/Dead fluorescence staining images of cells cultured in the different incubation groups. **(B)** Absorption at 450 nm in CCK-8 assay of different incubation groups. **(C)** Normalized CFU values of *E. coli* cells at different time points (0, 6 and 12 h). **(D)** Digital images showing the rabbit skin irritation at different testing duration. (*p < 0.01).

Typically, wearable flexible sensors in contact with the skin should have good biocompatibility. L929 cells were used as the model cell to investigate the biocompatibility of PU and LM@PU/CNT/LM membranes by *in vitro* experiments. Live/Dead fluorescence staining images showed a normal cell morphology in all groups (Figure 6A). As shown in Figure 6B, the absorption at 450 nm in the CCK-8 assay of all groups increased notably during the incubation time with a similar trend, indicating that there was no significant cytotoxic effect of the LM@PU/CNT/LM membrane.

E. *coli* cells were incubated on the PU and LM@PU/CNT/LM membranes to evaluate the antibacterial property of the membranes. Figure 6C illustrates the normalized CFU values of the *E. coli* cells at different time points (0, 6, and 12 h). Precisely, the ratio percentages of the CFU in the LM@PU/CNT/LM membrane group to the CFU in the control group (no membrane sample inside) were 47.20% ± 1.03% and 50.55% ± 0.83% after incubation for 6 h and 12 h, respectively, which were decreased with time. The ratio percentages of the CFU in the PU membrane group to the CFU in the control group were 89.49% ± 1.03% and 94.47% ± 1.50% after incubation for 6 h and 12 h, respectively. The Ga^3+^ in the solution released by liquid metal can permeate into the interior of *E. coli* cells and enhance the generation of reactive oxygen species (ROS). ROS is the single electron reduction product of oxygen which could induce oxidative damage to DNA and inhibit the division and proliferation of *E. coli* cells (Yang et al., 2020; Li L. et al., 2021; Qu et al., 2022). The CFUs of the LM@PU/CNT/LM membrane group were significantly lower than that in the other two groups, which proved that the LM@PU/CNT/LM membrane had an antibacterial effect.

Furthermore, the flexible substrate is composed of nanofibers prepared by electrospinning. The most remarkable properties of nanofibers are high porosity and good air permeability, which can provide sufficient space for gas exchange. So the porous electrospinning membrane has excellent permeability, which makes it suitable for wearable devices (Xu et al., 2020). In particular, air permeability and moisture permeability tests were performed on the LM@PU/CNT/LM membrane. The results showed that the LM@PU/CNT/LM membrane had high air permeability (6.10 mm/s) and moisture permeability (1.03 × 10^4^ g m^-2^·24 h), which were much higher than that of PDMS membrane (200 μm thick) the commercial medical patch (air permeability was about 0 mm/s, moisture permeability was below 50 g m^-2^·24 h) (Ma et al., 2021).

To further demonstrate that the flexible membrane applies to the skin surface, *in vivo* animal experiments on the epidermis of the depilated rabbit skin were performed. As seen in Figure 6D, the LM@PU/CNT/LM membrane did not cause obvious allergic irritation (that is, no erythema or oedema) on the rabbit skin surface during the observation periods, indicating that the LM@PU/CNT/LM membrane is skin-friendly. However, slight erythema was observed for the commercial gel patch 1 day after the test.

### 3.5 Monitoring of motion signals and ECG/EMG signals

**FIGURE 7:**
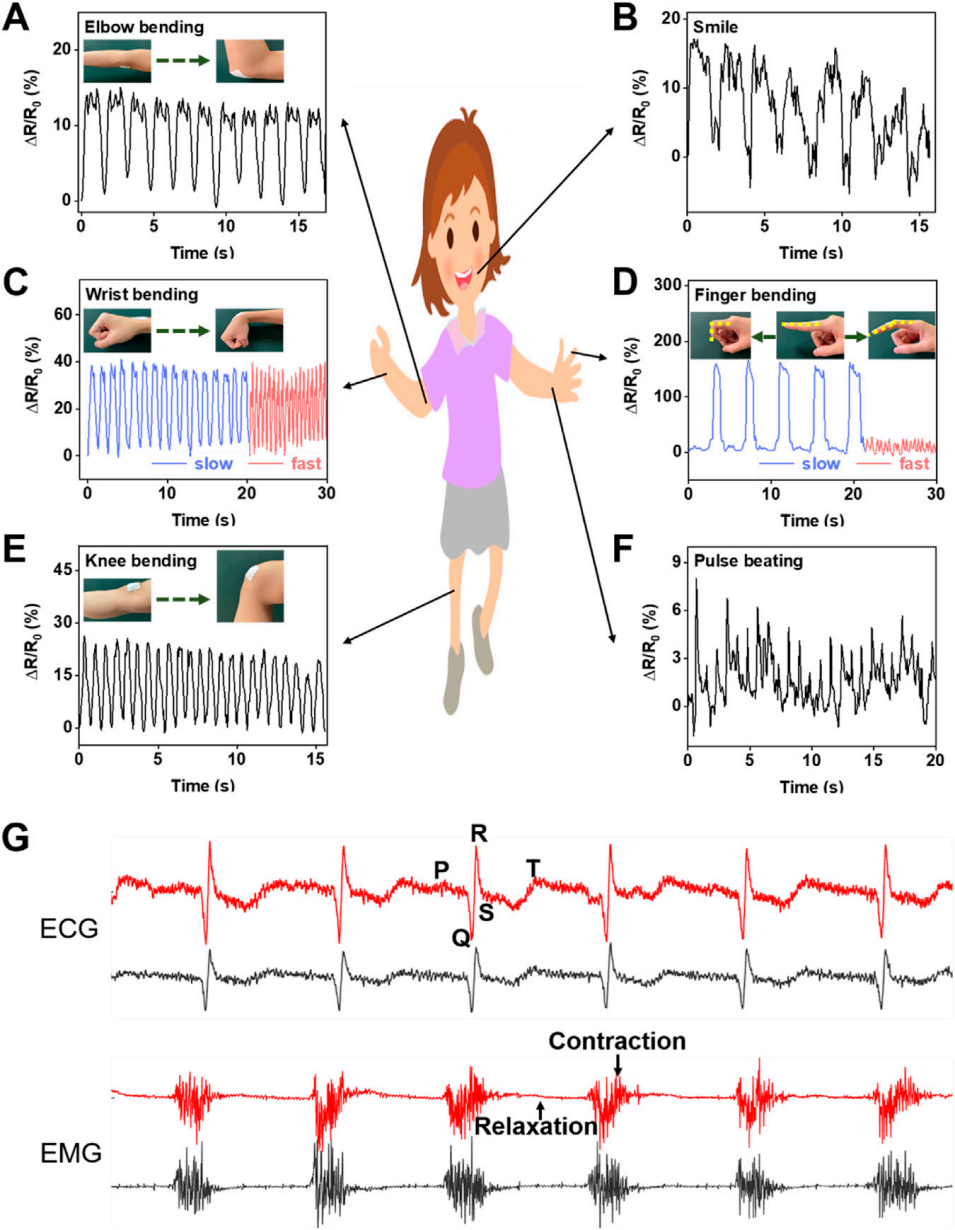
The LM@PU/CNT/LM flexible sensor was used to monitor the signal responses of various parts of the human body: **(A)** elbow bending, **(B)** smile, **(C)** wrist bending, **(D)** finger bending, **(E)** knee bending, **(F)** pulse beating, and **(G)** ECG and EMG signals detected using the LM@PU/CNT/LM membrane and commercially gel electrodes (red line - LM@PU/CNT/LM membrane; black line - commercially gel electrode).

The flexible membrane prepared by electrospinning technology has excellent wearability and can be easily contacted with skin. In addition, the membrane modified by LM and CNTs exhibits good electrical conductivity, high sensitivity, and good cyclic stability. Besides, it can be used as a wearable sensor to monitor various motion signals of the human body. The high permeability of the sensor provides good comfort for long-time wearing. Figures 7A–F shows the signal response of the LM@PU/CNT/LM flexible sensor for real-time monitoring of human motions. Specifically, the LM@PU/CNT/LM flexible sensor was contacted with the body, such as the elbow (Figure 7A), wrist (Figure 7C), finger (Figure 7D), and knee (Figure 7E), and its electrical resistance changed with the movement of the body. With the elbow, wrist, finger, and knee bending, the sensor is stretched and the electrical resistance increases. Meanwhile, as the elbow, wrist, finger, and knee being straight, the sensor returns to the initial state and the resistance decreases. Moreover, the sensor could sensitively distinguish the movements of the body parts when their moving frequencies and amplitudes were changed. The sensor could sensitively detect large, slow bends and small, fast bends of the finger (Figure 7D). After repeated bending-straightening-bending, the sensing signals remained almost constant, exhibiting good stability and repeatability. In addition to detecting large movements of the knee, elbow, finger, and wrist, the LM@PU/CNT/LM flexible sensor can also detect small strains such as a smile (Figure 7B) and a pulse beating (Figure 7F), showing promising applications in the field of wearable devices. Besides, the LM@PU/CNT/LM membrane can be used as a physiological electrical sensor to effectively monitor the ECG and EMG signals. As can be seen from Figure 7G, the ECG and EMG signals detected by the LM@PU/CNT/LM membrane are comparable to those of commercial gel patch electrodes. More so, clear characteristic waves (the P-wave, the QRS-wave and the T-wave) of the ECG and action potentials (contraction motion of muscles) of the EMG are successfully obtained, revealing the great application prospects of the LM@PU/CNT/LM membrane in health monitoring.

## 4 Conclusion

In this work, a LM@PU/CNT/LM membrane was obtained by coating LM onto a PU membrane composited with LMNPs and CNTs. Specifically, the membrane with point (LMNPs)-line (CNTs)-plane (LM thin-film) multiscale conductive structure has excellent mechanical strength (elongation at break of 490%; a tensile strength of 12 MPa) and high sensitivity (GF is 3.0 at 50% strain and 14.0 at 400% strain). Significantly, the LM@PU/CNT/LM membrane could effectively monitor human motion signals and physiological electrical signals, which is anticipated to be widely used in the field in health monitoring, soft robotics, and medical treatments.

## Data Availability

The original contributions presented in the study are included in the article/Supplementary Material, further inquiries can be directed to the corresponding author.
